# ASO Author Reflections: Modern-Day Implementation of Robotic Esophagogastric Cancer Surgery

**DOI:** 10.1245/s10434-021-11107-6

**Published:** 2021-11-27

**Authors:** Sivesh K. Kamarajah, Ewen A. Griffiths, Alexander W. Phillips, Jelle Ruurda, Richard van Hillegersberg, Wayne L. Hofstetter, Sheraz R. Markar

**Affiliations:** 1 Department of Upper Gastrointestinal Surgery, Queen Elizabeth Hospital Birmingham, University Hospitals Birmingham NHS Trust, Birmingham, UK; 2 Institute of Cancer and Genomic Sciences, College of Medical and Dental Sciences, University of Birmingham, Birmingham, UK; 3 Northern Oesophagogastric Unit, Royal Victoria Infirmary, Newcastle University Trust Hospitals, Newcastle-upon-Tyne, UK; 4 School of Medical Education, Newcastle University, Newcastle upon Tyne, Tyne and Wear, UK; 5 University Medical Center Utrecht, University Utrecht, Utrecht, The Netherlands; 6 Thoracic and Cardiovascular Surgery, The University of Texas MD Anderson Cancer Center, 1515 Holcombe Blvd, Houston, TX USA; 7 Department of Surgery and Cancer, Imperial College London, London, UK; 8 Department of Molecular Medicine and Surgery, Karolinska Institutet, Stockholm, Sweden

## Past

Over the past decade, there has been a marked increase in the uptake of robotic surgery in patients undergoing curative esophagectomy or gastrectomy for esophagogastric cancers. Although there is an expanding evidence base for minimally invasive techniques that appears to suggest either improved or similar morbidity, without compromising oncological quality,^1,2^ most of these studies preclude analysis comparing robotic esophagectomy or gastrectomy. To date, only one single-center European randomized controlled trial^3^ has shown improvements in postoperative complications, pain, short-term quality of life, and functional recovery when comparing robotic with open esophagectomy. Furthermore, a recent publication from the Upper Gastrointestinal International Robotic-assisted Association (UGIRA) demonstrated promising results of this technique when undertaken in high-volume specialized centers with adequate training.^4^ The data concerning robotic gastrectomy is largely based on observational cohort studies^5^ and originates from the Far East, with a different patient population and standard of lymphadenectomy to what is commonly observed in Western centers.^5,6^

## Present

The present study^7^ included patients with nonmetastatic esophageal and gastric cancers receiving open (esophagus, *n* = 11,442; stomach, *n* = 22,183), laparoscopic [esophagus (LAMIE), *n* = 4827; stomach (LAMIG), *n* = 6359], or robotic [esophagus (RAMIE), *n* = 1657; stomach (RAMIG), *n* = 1718] surgery from the US National Cancer Database (NCDB) (2010–2017). Patients receiving robotic surgery were more commonly treated within high-volume, academic centers and with advanced clinical T and N stage disease. From 2010 to 2017, textbook outcome (TO) rates increased for esophageal and gastric cancer treated by all surgical techniques. RAMIE [odds ratio (OR):1.41, CI_95%_: 1.27–1.58] and RAMIG (OR: 1.30, CI_95%_: 1.17–1.45) had significantly higher TO rates compared with open surgery. For esophagectomy, TO [hazard ratio (HR): 0.64, CI_95%_: 0.60–0.67] and RAMIE (HR: 0.92, CI_95%_: 0.84–1.00) were both associated with long-term survival. For gastrectomy, TO (HR: 0.58, CI_95%_: 0.56–0.60) and both LAMIG (HR: 0.89, CI_95%_: 0.85–0.94) and RAMIG (HR: 0.88, CI_95%_: 0.81–0.96) were all associated with long-term survival. Subset analysis in high-volume centers confirmed similar findings.

## Future

Moving forwards, dissemination of robotic surgery is key to ensuring routine adoption into clinical practice to optimize patient benefits. Firstly, implementation of training programs should be safe and be adopted within high-volume centers and/or surgeons to ensure a sufficient case volume to shorten any potential proficiency gain curve. Further, embedding adjuncts such as video-based analyses of performance, telemedicine for surgical coaching, and image-based surgery with projections of preoperative imaging may allow refined surgical anatomy and dissection in cancer surgery and shorten the learning curve among surgeons. Secondly, regulators and surgical community need to have highly regulated systems in place, such as international registries. This would be useful for (i) close monitoring of performance and uptake of robotic surgeries across various specialties and (ii) generating accurate data to inform the creation of appropriate safeguards; national bodies should consider providing coverage for robotic surgery with provisions for evidence development. The UGIRA was established to facilitate the reporting of robotic procedures worldwide and analyze variation and learning curves. Use of these provisions would facilitate greater understanding of how robotic procedures are being used in real-world practice. Akin to post-market surveillance of pharmaceuticals, such provisions would also create a common data resource from which the comparative safety and effectiveness of robotic operations can be evaluated by numerous investigators and is necessary for not only RAMIE/MIG, but also other types of surgery.

## References

[1] Mehta R, Paredes AZ, Tsilimigras DI (2020). Influence of hospital teaching status on the chance to achieve a textbook outcome after hepatopancreatic surgery for cancer among Medicare beneficiaries. Surgery..

[2] Van Boxel GI, Kingma BF, Ruurda JP (2020). Formal assessment of the learning curve for minimally invasive methods is vital in retrospective cohort studies. Am J Obstet Gynecol..

[3] van der Sluis PC, van der Horst S, May AM (2019). Robot-assisted minimally invasive thoracolaparoscopic esophagectomy versus open transthoracic esophagectomy for resectable esophageal cancer: a randomized controlled trial. Ann Surg..

[4] Kingma BF, Grimminger PP, van der Sluis PC (2020). Worldwide techniques and outcomes in robot-assisted minimally invasive esophagectomy (RAMIE): results from the multicenter international registry. Ann Surg..

[5] van Boxel GI, Ruurda JP, van Hillegersberg R (2019). Robotic-assisted gastrectomy for gastric cancer: a European perspective. Gastric Cancer..

[6] Ojima T, Nakamura M, Hayata K (2021). Short-term outcomes of robotic gastrectomy vs laparoscopic gastrectomy for patients with gastric cancer: a randomized clinical trial. JAMA Surg..

[7] Kamarajah SK, Griffiths EA, Phillips AW, et al. Robotic techniques in esophagogastric cancer surgery: an assessment of short- and long-term clinical outcomes. *Ann Surg Oncol*. 2021. 10.1245/s10434-021-11082-y 10.1245/s10434-021-11082-y PMC8989809 34890023

